# Reviewed article: Zago JKM, Anjos GM, McKenna GJ, Leles CR, Jordã LMR. Provision of dental prostheses in the oral health care network: a mixed methods study, Goiânia, Brazil, 2019. Epidemiol Serv Saude. 2026:35;e20250099

**DOI:** 10.1590/S2237-96222026v35e20250099.b

**Published:** 2026-03-27

**Authors:** Fernanda Campos de Almeida Carrer

**Affiliations:** 1 Universidade de São Paulo, São Paulo, SP, Brazil

## First round comments

Parabenizo pelo trabalho que pauta um assunto de extrema relevância para a RASB que se forma a partir de 2024 no Brasil, entretanto, de acordo com parecer os revisores fica clara a necessidade de uma grande revisão antes que possamos definir o real potencial de publicação do manuscrito. O método precisa ser reescrito, a introdução não delimita o problema e a conclusão avança para além dos resultados apresentados e é necessária uma revisão completa e novos pareceres antes de uma decisão. Estamos certos que o esforço de adequar o texto vai melhorar o manuscrito e que podemos ajudar neste processo.

Esperamos a versão revisada o mais breve possível

Recommendation: Major revision

## Second round comments

Os senhores fizeram todas as sugestões apontadas pelos revisores e o texto melhorou muito em qualidade. Eu ainda gostaria de ver uma discussão que dialogasse mais com o SUS, Brasil Sorridente e contexto dos municípios, especialmente com a Lei nº 14.572/23, que a incluiu na Lei Orgânica da Saúde, tornando a saúde bucal um direito de todos. Faltou falar mais de rede, de contribuir com o tomador de decisão. Eu sugiro mais uma revisão, com foco na discussão!

Recommendation: Minor revision

## Third round comments

Obrigada pelo retorno, mas infelizmente não é possível aceitar a versão R1, tendo em vista que as sugestões dos revisores não foram consideradas e atendidas. O texto precisa ser revisto, excluindo trechos redundantes e acrescentando as preciosas sugestões dos revisores. Sabemos da limitação de caracteres, mas os pareceres deixam claro diversas alterações que precisam ser observadas.

Não é possível afirmar se o manuscrito poderá ser aceito, porque a versão corrigida é muito semelhante à versão original e preserva elementos que carecem de revisão.

Espero que os autores façam as alterações, pontuando no texto tudo que foi acrescentado e suprimido, no próprio texto e na carta resposta, que deve trazer resposta a cada item apontado pelos revisores.

Um abraço e bom trabalho

Recommendation: Major revision

